# Direct, Indirect, and Buffering Effects of Support for Mothers on Children’s Socioemotional Adjustment

**DOI:** 10.1037/fam0000438

**Published:** 2018-08-09

**Authors:** Alison Parkes, Helen Sweeting

**Affiliations:** 1 MRC/CSO Social and Public Health Sciences Unit, University of Glasgow

**Keywords:** social support, health and social work professional support, parenting, externalizing problems, internalizing problems

## Abstract

Support for mothers may improve children’s socioemotional adjustment, yet few studies have considered the benefits of formal support (from health and social work professionals) in addition to social support (from family and friends) or explored the mechanisms. These issues were addressed using a birth cohort (*n* = 2,649) to explore how mothers’ perceptions of social and formal support when children were ages 10–22 months predicted trajectories of children’s externalizing and internalizing problems from 58 to 122 months. We tested mediating pathways from support to child adjustment via 3 family stressors measured at 46–58 months (maternal distress, economic strain, and dysfunctional parenting) and examined whether support buffered effects of stressors on child adjustment. Social and formal support were simultaneously associated with lower child externalizing and internalizing problem trajectory intercepts at 90 months but did not predict trajectory slopes. Social support effects were mediated mainly via lower maternal distress, which then reduced children’s problems via lower dysfunctional parenting, or more directly. Additional indirect effects involved lower economic strain. Formal support effects were mediated to a lesser extent by reduced dysfunctional parenting. Two buffering effects were found: social support reduced effects of economic strain on internalizing problems, and formal support reduced effects of dysfunctional parenting on internalizing problems. Findings suggest measures promoting families’ social integration should benefit children’s socioemotional adjustment via improved parental psychological and economic resources and by buffering impacts of economic strain. Enhancing access to health and welfare services through greater awareness and trust should benefit children’s adjustment, via improved parenting and by buffering impacts of dysfunctional parenting.

In middle childhood, externalizing problems (aggression, rule breaking, and attentional problems) signal a risk of antisocial and health risk behavior, poor mental health, and low academic attainment in adolescence and young adulthood, whereas the emergence of internalizing problems (depressive and anxiety symptoms, somatic complaints, and withdrawal) is associated with later depression (Fergusson, Horwood, & Ridder, 2005; Sayal, Washbrook, & Propper, 2015; Weeks et al., 2016). Ecological and process models highlight the pivotal role of within-family processes for children’s socioemotional adjustment, together with the contribution of extrafamilial resources from the wider community, toward shaping these processes (Belsky, 1984; Bronfenbrenner & Morris, 1998). In practice, research attention directed at understanding children’s adjustment problems has focused almost exclusively on within-family processes, largely neglecting the role played by extrafamilial support for parents (McConnell, Breitkreuz, & Savage, 2011). Moreover, few studies assessing benefits of extrafamilial support for children have acknowledged the need to discount several alternative possibilities (Ryan, Kalil, & Leininger, 2009). Families with heightened risk of child adjustment problems (e.g., those living in poverty, where mothers are depressed or where infants have developmental problems) may be less able or predisposed to draw on support from others, have a negative bias in reporting family circumstances, and/or have complex support needs that are inherently less likely to be fulfilled. To our knowledge, only one study (Ryan, Kalil et al., 2009) has allowed for a sufficiently wide range of endogenous maternal and child characteristics to enable us to discount selection effects and reporting bias. This study was confined to the effect of social support in two low-income samples from the United States and did not explore potential mechanisms.

Additional rigorous studies are needed to consider benefits of formal support for mothers from professional health and welfare services, as well as social support, and to investigate pathways linking support to child outcomes. Observational studies of formal support in relation to child adjustment are currently sparse (Leventhal, Brooks-Gunn, McCormick, & McCarton, 2000; Spielberger & Lyons, 2009) and do not consider mechanisms. Greater clarity in relation to underlying mechanisms would increase confidence in causal effects of both social and formal support for mothers on children’s adjustment. Family stress and ecobiodevelopmental models point to the harmful effect of within-family stressors on children’s adjustment (Conger, Ge, Elder, Lorenz, & Simons, 1994; Shonkoff, Garner, the Committee on Psychosocial Aspects of Child and Family Health, Committee on Early Childhood, Adoption, and Dependent Care, & the Section on Developmental and Behavioral Pediatrics, 2012). This suggests that one should examine how support may alleviate such stressors. In particular, there should be exploration of whether support reduces and/or buffers the effects of dysfunctional parenting (characterized by high levels of negativity, parenting stress, and a chaotic home environment), together with economic hardship and poor maternal mental health. All these have been widely implicated in children’s socioemotional adjustment (see, e.g., Berg-Nielsen, Vikan, & Dahl, 2002; Hur, Buettner, & Jeon, 2015; Kiernan & Mensah, 2009; Östberg & Hagekull, 2013).

This study examines effects of social and formal support for mothers on child socioemotional adjustment, using a nationally representative sample from the United Kingdom. It assesses whether any beneficial effects of support are mediated by reduced within-family stressors and whether support buffers the impact of these stressors on child adjustment.

## Defining Social and Formal Support

In common with other researchers examining social support from family and friends (Bonds, Gondoli, Sturge-Apple, & Salem, 2002; Choi & Pyun, 2014; Heberle, Krill, Briggs-Gowan, & Carter, 2015; Herwig, Wirtz, & Bengel, 2004; C.-Y. S. Lee, Anderson, Horowitz, & August, 2009; McConnell et al., 2011; Östberg & Hagekull, 2013; Ryan, Kalil et al., 2009), we did not examine enacted (received) support when assessing benefits for child adjustment. This is because of the difficulty in distinguishing preventive support (which may predict better outcomes) from responsive support that is related to need (and consequently often associated with poorer outcomes, especially in cross-sectional analyses). For *social support,* we followed others cited by drawing on two overlapping constructs: “social embeddedness” (number and quality of social ties) and “perceived support” (availability and adequacy), both presumed to signal a parent’s ability to draw on actual support from family and friends (Barrera, 1986, p. 415).

Observational studies of the impact of *formal support* for parenting from professional health and welfare services on child adjustment are inconclusive, reflecting reliance on measures of enacted support likely to be conflated with family need (Leventhal et al., 2000; Spielberger & Lyons, 2009). To measure formal support, we adopted a similar approach to that commonly taken for social support, using parental perceptions of availability and adequacy. Despite widespread provision of family support services in many Western countries, many parents perceive barriers to access (Ghate & Hazel, 2002; Whittaker & Cowley, 2012). Barriers partly relate to low awareness or practical problems surrounding travel, opening hours, or administrative procedures. It is important to note, however, that they also relate to perceptions that available support is inadequate to meet needs and to fears of interference and stigma.

## Pathways From Support to Child Socioemotional Adjustment

**Figure 1 fig1:**
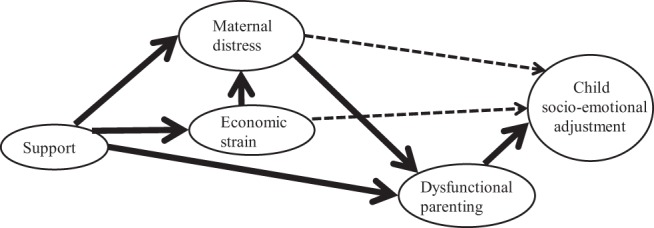
Conceptual model depicting paths from support for mothers to child socioemotional adjustment via maternal distress, economic strain and dysfunctional parenting. Solid arrows show hypothesized main paths, and broken arrows show potential additional paths.

This study tests pathways from support for mothers to children’s socioemotional adjustment via three family stressors: dysfunctional parenting, maternal distress, and economic strain (see Figure 1). In this model, most hypothesized effects of support involve reduced dysfunctional parenting. Social embeddedness has long been conceived as having direct benefits for parenting, via mechanisms such as provision of information and advice, modeling of appropriate behavior, and positive affirmation of a parent’s own attitudes and parenting skills (Belsky, 1984, 1990; McConnell et al., 2011). Perceived availability of formal support might have similar benefits, signaling greater engagement with universal provision of professional information and advice to parents that increases their knowledge and skills. Empirical evidence that parenting quality is linked to support availability, however, has been relatively sparse and confined to studies of social support (Bonds et al., 2002; C.-Y. S. Lee et al., 2009). Existing studies of young families’ service use have yielded few conclusions on benefits for parenting (Maupin, Brophy-Herb, Schiffman, & Bocknek, 2010; Spielberger & Lyons, 2009). This may reflect a cross-sectional design, coupled with measures of *enacted* support. Further work is therefore needed to substantiate a path from support to adjustment via parenting alone, as shown in our conceptual model (see Figure 1).[Fig-anchor fig1]

Parenting process and family stress models (Belsky, 1984; Conger et al., 1994) highlight the importance of parental psychological and economic resources for effective parenting. This points to further paths whereby support could reduce maternal distress and economic strain, both of which in turn affect adjustment via improved parenting (see Figure 1). Social embeddedness provides families with opportunities for emotional and instrumental support and is thought to be important in regulating maternal psychological functioning, even without any major stressors (Barrera, 1986; Cohen & Wills, 1985; Thoits, 2011). Emotional support from family and friends may enable parents to share frustrations over minor daily hassles with a sympathetic audience, thereby preventing further escalation of problems (Lakey & Orehek, 2011). Instrumental support from family and friends may also reduce economic strain (Harknett, 2006), which may sustain maternal psychological functioning indirectly (Conger et al., 1994). Although little is known about the possibility, conceivably mothers’ access to professional services could provide similar sustaining emotional and instrumental support.

Although benefits of social support for mothers’ psychological well-being are well established (see, e.g., Brown, Harris, Woods, Buman, & Cox, 2012; Manuel, Martinson, Bledsoe-Mansori, & Bellamy, 2012), only a few studies have explored complete pathways to child adjustment via these constructs (Choi & Pyun, 2014; Herwig et al., 2004; Östberg & Hagekull, 2013). Two were cross-sectional in design, so one cannot be sure about the direction of effects. One longitudinal study found that reduced economic strain, as well as lower parenting stress and better parenting, mediated effects of greater perceived social support on young children’s behavior problems (Choi & Pyun, 2014). However, it contained no explicit measurement of maternal psychological functioning and did not adjust for possible confounders of support−child outcome associations.

Additional pathways from social support not involving parenting (see Figure 1, dashed arrows) might impact both externalizing and internalizing problems. Maternal distress may model dysregulated behaviors and emotions (Heberle et al., 2015), whereas economic strain could lead to greater adjustment problems, even in young children, via negative social comparisons and poverty-related stigma (Heberle & Carter, 2015).

## Buffering Effects of Support

Social support has long been theorized to have a protective or buffering effect on psychological functioning, by offering appraisal and/or coping mechanisms to deal with stressors (Cohen & Wills, 1985). Although buffering was originally conceived in relation to adult psychological functioning, it is possible to extend this idea in relation to children’s socioemotional adjustment. The availability of support may protect children from the harmful effects of disruptive family processes, if parents are able to draw on support for appraisal−coping strategies, and/or if other individuals directly offer the child appraisal−coping strategies that dilute or counteract family stressors. Empirical evidence in relation to child socioemotional adjustment has been limited to studies investigating moderating effects of social support on stressors such as maternal depression and suboptimal parenting. These present mixed findings. In two studies, support buffered effects of lower maternal psychological well-being and suboptimal parenting on children’s externalizing or internalizing behavior problems (Barnett, Scaramella, Neppl, Ontai, & Conger, 2010; Heberle et al., 2015). In contrast, another study found support was less, rather than more, effective in protecting children from severe maternal depression (L.-C. Lee, Halpern, Hertz-Picciotto, Martin, & Suchindran, 2006), whereas two others found no moderating effects of social support on the effects of parenting stress (McConnell et al., 2011; Östberg & Hagekull, 2013; Ryan, Tolani, & Brooks-Gunn, 2009). Although not previously explored, perceived availability of formal support may have a similar protective role in times of stress. It may signal access to assistance with parenting problems, as well as access to other forms of support relieving parental psychological distress and economic strain, such as couple relationship counseling, substance abuse treatment, or access to welfare payments. Further research is needed to establish whether formal, as well as social, support generally has protective buffering effects in relation to children’s adjustment or whether its action is compromised by contextual strains such as maternal depression.

## Study Hypotheses

Our study explores pathways from support for mothers to two aspects of children’s socioemotional adjustment (externalizing and internalizing behavior problems) via three family stressors in our conceptual model: maternal distress, economic strain, and dysfunctional parenting. We also explore whether support moderates associations between the stressors and child adjustment. We aimed to test the following hypotheses:

*Hypothesis 1:* Both social and formal support will predict better child socioemotional adjustment.

*Hypothesis 2:* Positive effects of both types of support will be mediated via less economic strain, maternal distress and dysfunctional parenting.

*Hypothesis 3:* Both types of support will have buffering effects in reducing the impact of economic strain, maternal distress, and dysfunctional parenting on child adjustment.

Based on existing literature, we put forward similar hypotheses in relation to both aspects of child adjustment studied.

To strengthen causal inference, our study has a prospective design with social and formal support for mothers measured in the vulnerable early years of children’s lives (infancy and toddlerhood, 10–22 months) and before behavioral problems are likely to develop. We examined the influence of support on trajectories of socioemotional adjustment in middle childhood (measured from approximately ages 6 to 10), because as noted this signals adolescent and young adult risk. Potential mediators are measured at an intermediate time point (ages 4–5). To help overcome potential hazards associated with selection effects and maternal bias, our analyses adjusted for a wide range of confounders measured in infancy that are associated with support, mediators, and outcome variables, including baseline maternal mental health and socioeconomic information.

## Method

Data were from the Growing Up in Scotland study’s first birth cohort (children born 2004–2005; further details available in Bradshaw, Tipping, Marryat, & Corbett, 2007). Baseline data were gathered from 5,217 families in 2005–2006, when children were 10 months old. Families were followed up annually for 5 years (to 70 months) and then at approximately two-year intervals (94 and 122 months). Each data collection sweep was subject to medical ethical review (Scotland “A” Multi Research Ethics Committee), with mothers or caregivers giving informed consent.

This study used data from computer-assisted personal interviews with the main caregiver. We excluded 93 families with multiple births and a further 103 families where the main caregiver interviewed at child age 10 and 22 months was not the natural mother. Of the remaining 5,021 families, 3,598 (72%) were followed up at child age 46 and 58 months. We further excluded 62 families where the child’s natural mother was not the main caregiver interviewed about potential mediators at these ages, giving an eligible sample of 3,536 families. Of these, 3,031 families (86%) were followed to the final time point. To provide consistent reporting of child outcomes, we restricted the analysis sample to cases where the mother was interviewed at all relevant outcome time points (70, 94, and 122 months) and provided outcome information on at least one of these occasions (*n* = 2,649; 87% of the complete eligible sample follow-up). The analysis sample contained fewer mothers with low support and low educational qualifications compared to a complete follow-up of the eligible sample but did not differ regarding other covariates.

### Measures

All measures were based on information supplied by the child’s natural mother.

#### Main child outcomes: Socioemotional adjustment

Adjustment was measured at 70, 94, and 122 months using the Strengths and Difficulties Questionnaire (R. Goodman, 1997). Items ask for agreement with statements concerning the child, with response options rated on this 3-point scale: 0 (*not*), 1 (*somewhat*), and 2 (*certainly true*). Scores are nonnormally distributed (Stone, Otten, Engels, Vermulst, & Janssens, 2010). *Externalizing problems* used the combined conduct problems and hyperactivity−inattention five-item subscales (Cronbach alphas at each age = .74–.80); *internalizing problems* used the combined peer relationship and emotional problems five-item subscales (Cronbach alphas = .61–.76). Externalizing and internalizing scores have good convergent and discriminant validity across informants and with respect to clinical disorder (A. Goodman, Lamping, & Ploubidis, 2010).

#### Support measures

A factor analysis of all support items used in this study found a two-factor solution, with all items described for social support loading onto one factor (loadings = .5–.7) and all items described for formal support (including two items that do not make specific reference to professional services) loading onto the second factor (loadings = .4–.6).

#### Social support

A standardized scale (Cronbach’s alpha = .65) was created from four items, measured at 22 months, concerning support for mothers from family and friends. The first item asked: “Not counting people who live with you, which of the following statements best describes how many people you have a close relationship with?” rated on this 4-point scale: 1 (*I have close relationships with lots of people*), 2 (*I have close relationships with some people*), 3 (*I have one or two close relationships*), and 4 (*I don’t have any close relationships*). The second asked: “Thinking about your immediate family (parents and brothers or sisters) living elsewhere, can you tell me how much you agree or disagree with the following statement: ‘I feel close to most of my family’?” rated on a 5-point scale ranging from 1 (*agree strongly*) to 5 (*disagree strongly*). The third asked for agreement with the statement “My friends take notice of my opinions,” rated on a 5-point scale ranging from 1 (*agree strongly*) to 5 (*disagree strongly*); mothers reporting no family (*n* = 9; .3%) or no friends (*n* = 25; 1.2%) were recoded as *disagree strongly.*^1^ Last, mothers were asked: “Overall, how do you feel about the amount of support or help you get from family or friends living elsewhere?” rated on a 3-point scale ranging from 1 (*I get enough help*) to 3 (*I don’t get any help at all*^2^).

#### Formal support

A standardized scale (Cronbach’s alpha = .64) used six items administered at 10 and/or 22 months (one item was presented at both ages) from a previous study of support among low-income families (Ghate & Hazel, 2002) that asked mothers’ agreement with statements concerning parenting advice available from professionals such as health visitors. Responses were rated on a 5-point scale ranging from 1 (*strongly agree*) to 5 (*strongly disagree*). The item administered at both ages was “If you ask for help or advice on parenting from professionals like doctors or social workers, they start interfering or trying to take over.” Those at 10 months only were “It’s difficult to ask people for help or advice about parenting unless you know them really well” and “It’s hard to know who to ask for help or advice about being a parent.” Those at 22 months only were “Professionals like health visitors and social workers do not offer parents enough advice and support with bringing up their children” and “If other people knew you were getting professional advice or support with parenting, they would probably think you were a bad parent.”

To validate the social support measure, we examined associations with instrumental support received when the child was age 34 months. Mothers perceiving high social support were more likely than mothers reporting low social support to have weekly grandparental child care for an hour or more (72% vs. 32%, *p* < .001) and to receive grandparental weekly help with household chores or purchases (42% vs. 27%, *p* < .001). It was not possible to validate attitudes to formal support against later receipt. However, it is important to note that attitudes are unlikely to be based solely on prejudice and hearsay, because all mothers received routine universal postnatal support from health visitors referred to in the interview items. Attitudes were also associated with use and perceptions of universally available antenatal support measured in the survey (details are available on request).

#### Family stressors

*Maternal distress* measured at 46 and 58 months used factor scores of two indicators (both loadings = .7): These were the combined depression and stress subscales from the short form of the Depression Anxiety and Stress Scale (Henry & Crawford, 2005) at 46 months and the mental health subscale from the Short Form Health Survey (SF-12; Jenkinson & Layte, 1997). *Economic strain* at 46 and 58 months used factor scores (loadings = .6–.9) of four items: one concerning unaffordability of 10 common household necessities from a European material deprivation score (European Union, 2012) and three indicators of money problems at 58 months: the number of unpaid household bills based on 13 common items; difficulty repaying debts, rated on a 4-point scale ranging from 1 (*almost all the time*) to 4 (*never*); and rating of family financial management on this 6-point scale: 1 (*Manage very well*), 2 (*Manage quite well*), 3 (*Get by alright*), 4 (*Don’t manage very well*), 5 (*Have some financial difficulties*), and 6 (*Are in deep financial trouble*). *Dysfunctional parenting* used factor scores (loadings = .6–.7) of three indicators at 58 months: parenting stress (four items from the Parental Stress Scale; Berry & Jones, 1995; Cronbach’s alpha = .71), mother−child conflict (eight items from the Pianta scale; Pianta, 1992; Cronbach’s alpha = .82), and home disorganization (three items from the Confusion, Hubbub, and Order Scale; Matheny, Wachs, Ludwig, & Phillips, 1995; Cronbach’s alpha = .67).

#### Covariates

The covariates included child, maternal, and household characteristics identified in the literature as potential confounders of associations between support, mediators, and outcomes, including those used in a previous study (Ryan, Kalil et al., 2009), to discount the possibility of bias and selection effects. They were measured at 10 months (unless otherwise stated). *Child characteristics* comprised sex and developmental delay, assessed at 22 months using the Communication and Symbolic Behavior Scales Developmental Profile (Wetherby, Allen, Cleary, Kublin, & Goldstein, 2002) and applying the recommended cutoff. *Maternal characteristics* comprised age, ethnic minority, educational level, smoking during pregnancy, partner relationship quality (based on four items at 22 months, standardized α = .76 from the Golombok Rust Inventory of Marital State; Rust, Bennun, Crowe, & Golombok, 1990), and mental and physical health using the SF-12 subscales (Jenkinson & Layte, 1997). *Household characteristics* comprised measures of composition (presence of the child’s father, one or more grandparents and any other adults, number of children) and poverty (based on a score of three indicators each measured at both 10 and 22 months: household income <60% of median United Kingdom income, neither resident parent in paid employment, and receipt of means-tested benefits [income support, housing benefit, council tax benefit]). Descriptive statistics for covariates are provided in Resource 1 of the online supplemental materials.

### Analysis

Multivariable models used Mplus Version 7.3 (Muthén & Muthén, 1998–2012). Missing data for individual items was generally low (<1%). Incomplete information was predicted by the mother’s having educational qualifications below the level of Scottish Highers (school-leaving university entrance qualifications), speaking a language other than English at home, not living with the child’s father, and a grandparent living in the household. A complete case analysis would have resulted in loss of 13% of the eligible sample, with the risk of bias. To reduce bias and increase statistical power, we imputed missing item responses using the Mplus multiple imputation facility. Inclusion of all variables predicting missingness in the imputation model increased the plausibility of the missing at random assumption. Analyses used results pooled across 20 imputed data sets, took account of the complex survey design, and used survey weights to counteract differential attrition. To address nonindependence of observations in the complex sample and nonnormality of measures, we used maximum likelihood estimation with robust standard errors computed using a sandwich estimator. To permit comparison of effect sizes and aid interpretation of interactions, main exposure measures and mediators were all standardized.

Children’s externalizing and internalizing problems were modeled as parallel latent growth processes from 70 to 122 months (approximately six–ten years), with intercepts set at 90 months (7.5 years). A multivariable model examined associations between the two maternal support measures and adjustment trajectories, adjusting for covariates. Next, three stressors (maternal distress, economic strain, and dysfunctional parenting) acting as potential mediators of maternal support–child problem associations were added in stages to create a path model, using the conceptual model as a guide. Comparative fit of models with different sets of indirect pathways was assessed using the Akaike and Bayesian information criteria (AIC and BIC, respectively), with smaller values indicating better fit. Cutoffs applied to assess absolute fit were <.06 for the root-mean-square error of approximation (RMSEA) and <.08 for the standardized root-mean-square residual (SRMR; Hu & Bentler, 1999). Indirect effects from maternal support to outcomes via stressors were calculated in the final path model using the Mplus model indirect facility, with bias-corrected bootstrap standard errors computed following recommended practice (MacKinnon, Lockwood, & Williams, 2004). Last, moderation of stressor−outcome associations was tested by adding Maternal Support × Stressor interaction terms to the path model. Throughout, statistical significance was defined at the *p* < .05 level.

Sensitivity analyses based on data sets with (a) complete case information and (b) complete information on independent variables, with missing information for dependent variables handled using full information maximum likelihood, gave closely similar findings to those using imputed data. We report results using imputed data here, except for results for indirect effects: Due to software constraints on bootstrapping, these were produced using the latter data set.

## Results

Correlations between measures of maternal support, child externalizing and internalizing problems, and potential mediators were generally small to moderate in size (see Resource 2 of the online supplemental materials, which also provides descriptive statistics for these measures).

The unconditional latent growth curve model found that mean trajectories of child externalizing problems declined over the study period, whereas internalizing problems increased (for a graph, see Resource 3 of the online supplemental materials). To test Hypothesis 1, that both social and formal support would predict child adjustment, we allowed support and covariates to predict all growth terms. Model fit was satisfactory (RMSEA = .03, SRMR = .01). Higher social and formal support independently predicted lower externalizing and internalizing trajectory intercepts (see Table 1). Effect sizes reported are standardized with respect to predictors (thus, e.g., a 1-*SD* increase in mothers’ social support predicted a .23-point reduction in the externalizing problem intercept). Except for a small association between formal support and the externalizing quadratic term, support did not predict linear or quadratic terms (i.e., support did not predict changes in problems over time). There was no interaction between the two support measures (not shown).[Table-anchor tbl1]

**Figure 2 fig2:**
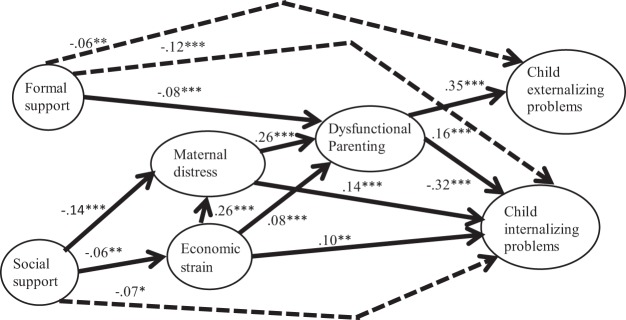
Final path model. Solid arrows represent indirect paths from support at 10–22 months to child adjustment trajectory intercepts (90 months) via mediators (46–58 months). Dashed arrows represent direct paths. For ease of comparison of pathways, figures represent coefficients standardized with respect to predictors *and* outcome. For simplicity, the figure omits nonsignificant associations. Support−mediator and support−outcome associations are adjusted for child gender and developmental delay, maternal age, ethnic group, education, physical and mental health, smoking in pregnancy, couple relationship, father in household, number of children, grandparent in household, additional adult in household, and family poverty. * *p* < .05. ** *p* < .01. *** *p* < .001.

To test Hypothesis 2, relating to mediation, we created a path model based on the conceptual model, testing comparative model fit in stages using AIC and BIC values. A model corresponding to the full conceptual model in Figure 1 provided the best fit, compared to subsets of this model. Absolute fit of this final path model was also satisfactory (RMSEA = .03, SRMR = .01). Table 2 shows the effect of support on trajectory intercepts at 90 months before and after adjusting for mediators (note that, although not shown in this table, there was no effect of mediators on the small association found for the externalizing quadratic term). Mediators attenuated effects of social support by 61% (externalizing problems) and 42% (internalizing problems), with the direct effect of social support on externalizing problems no longer significant. Mediators produced relatively weak attenuation of formal support effects (34% externalizing, 13% internalizing), with direct effects remaining significant. Figure 2 shows the final path model (note that unlike the case in Table 2, Figure 2 coefficients are standardized with respect to outcomes as well as predictors).[Table-anchor tbl2][Fig-anchor fig2]

Table 3 provides estimates of significant indirect effects of support on trajectory intercepts, with bias-corrected bootstrapped 95% confidence intervals. Hypothesis 2, concerning mediating pathways involving all three family stressors, was confirmed for social support only. The largest pathway from social support to externalizing problems was via maternal distress and dysfunctional parenting. The two largest pathways from social support to internalizing problems were via maternal distress only and via both maternal distress and dysfunctional parenting. Only dysfunctional parenting mediated effects of formal support, with a larger indirect pathway to externalizing than to internalizing problems.[Table-anchor tbl3]

**Figure 3 fig3:**
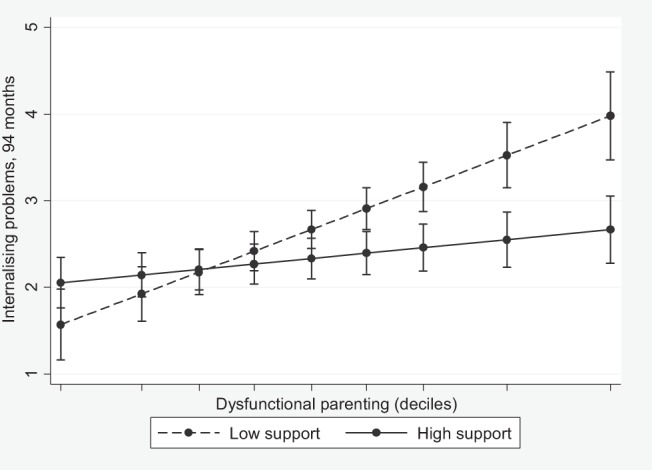
Moderating effect of formal support on association between dysfunctional parenting and children’s internalizing problems. The figure shows the linear prediction of mean internalizing problems at 94 months (with error bars indicating 95% confidence intervals), according to the level of dysfunctional parenting at 58 months. High and low support lines represent the highest and lowest deciles of positive attitudes toward formal support (10–22 months). Prediction is conditional on child gender and developmental delay, maternal age, ethnic group, education, physical and mental health, smoking in pregnancy, couple relationship, father in household, number of children, grandparent in household, additional adult in household, family poverty, and social support.

To investigate Hypothesis 3, concerning buffering effects of support on the three stressors, we allowed Support × Stressor interaction terms to predict adjustment trajectory intercepts. No moderating effect was found in relation to externalizing problems. Economic strain and dysfunctional parenting both predicted a higher internalizing intercept (respectively, β = .18, *p* = .013, and β = .36, *p* < .001). Social support moderated effects of economic strain (β = −.12, *p* = .027), whereas formal support moderated effects of dysfunctional parenting (β = −.22, *p* = .009). Figure 3 illustrates the larger of these two effects, indicating that perceived formal support had a greater protective effect on children’s internalizing problems at higher levels of dysfunctional parenting.[Fig-anchor fig3]

## Discussion

In this large, representative sample, lower (perceived) levels of social support and more negative attitudes toward formal support among mothers of infants and toddlers predicted lower levels of school-age socioemotional adjustment. The study extends previous findings of an association between availability of social support and young children’s socioemotional adjustment in two low-income samples from the United States (Ryan, Kalil et al., 2009) to a different (United Kingdom general population) setting, using a similar range of robust controls for endogenous maternal, child, and family characteristics. Our study makes an important additional contribution, in showing that perceived formal support from health and social work professionals was also associated with children’s socioemotional adjustment. Although we were not able to explore mothers’ subsequent engagement with support services, negative perceptions are known to deter parents from using family support services and participating in parenting programs (Ghate & Hazel, 2002; Whittaker & Cowley, 2012).

This study also contributes to an understanding of pathways from two sources of maternal support to children’s adjustment. Effects of social support were mediated mainly via reduced maternal distress, confirming the role of social support in sustaining mothers’ psychological functioning found in much previous work (see, e.g., Brown et al., 2012; Manuel et al., 2012). We have additionally shown that the effects of social support on maternal distress then decreased children’s problems via reduced dysfunctional parenting or (in the case of internalizing problems) more directly. We also found a weaker pathway from social support via reduced economic strain, strengthening another study that did not allow for maternal distress (Choi & Pyun, 2014). The effects of social support were not, however, transmitted via parenting alone. This appears to counter some previous work finding direct effects of social support on parenting, although without (as here) testing a complete pathway to child adjustment (Bonds et al., 2002; C.-Y. S. Lee et al., 2009). In contrast, effects of formal support on child adjustment were, in part, attributable to a direct effect of formal support on parenting. This may reflect a link between perceived support and mothers’ motivation and capacity to seek advice on parenting from professional sources, as well as greater receptivity to professional expertise. Our findings extend previous longitudinal work on social support only (Choi & Pyun, 2014; Heberle et al., 2015; Herwig et al., 2004) by allowing for alternative mediators and more extensive baseline confounders.

Our study also found that both social and formal support had buffering effects on stressors associated with children’s internalizing problems. To our knowledge, buffering of economic strain has not been reported elsewhere. When household resources are limited, supplementary financial or in-kind provision may alleviate children’s feelings of sadness and anxiety, as well as reduce stigma associated with poverty (Heberle & Carter, 2015). Buffering of dysfunctional parenting by formal support might involve specialized assistance with circumstances compromising a mother’s parenting capabilities, such as provision of skills to manage children’s behavior, or access to child care enabling relief from child-rearing responsibilities. Specialized services might also directly protect the child, via help in coping with negative feelings and building resilience. Nonetheless, we did not find a moderating effect of social support on the effects of dysfunctional parenting, in contrast to two other studies (Barnett et al., 2010; Heberle et al., 2015); this could reflect measurement differences and/or context.

Our study has several limitations, notably reliance on information from mothers. This neglects the perspectives of fathers and other caregivers and also risks inflated associations between support and outcomes from common method variance. Although a range of covariates helped discount the possibility of selection effects, we cannot discount the possibility of omitted confounders. There are some additional threats to a causal interpretation of our findings. Support predicted the overall *level* of child adjustment but did not predict *change* in child adjustment. This suggests a need to assess child adjustment at an earlier time, to establish whether its development is affected by support. Other threats stem from simultaneous measurement of mediators, overlap between measurement of mediators and the start of adjustment trajectories, and the finding that mediators did not explain all associations between support and outcomes. Further work would benefit from the availability of repeated main measures, using these in fixed effects and cross-lagged models, and from investigation of additional mediators such as positive parenting.

Despite shortcomings, the study has several strengths. It uses a large sample representative of the Scottish population at baseline, reducing the risk of bias through use of survey weights and multiple imputation of missing information. This increases generalizability of study findings, although our study population had low representation of certain risk groups such as (a) lone mothers without educational qualifications and (b) migrants. Future research should address these groups and attend to measurement issues. It is difficult to make firm comparisons between the two sources of support in our study. Our social support measure largely reflected social embeddedness, which although likely to encompass emotional and instrumental support may not always signal responsiveness to need (Cohen & Wills, 1985). In contrast, our measure of formal support, based on perceptions of availability and adequacy, may more accurately have reflected mothers’ ability and willingness to draw on support when required. It is also difficult to make precise comparisons with other cited studies, which have used a wide range of support measures reflecting emotional and/or instrumental support to varying extents. In the future, greater consistency of measurement is desirable to distinguish the influence of population group and/or context.

In conclusion, our study adds to existing evidence on the importance of social ties outside the immediate family (Cuellar, Jones, & Sterrett, 2015; Marshall, Noonan, McCartney, Marx, & Keefe, 2001), suggesting the benefits of promoting the social integration of young families for children’s socioemotional adjustment and pathways through which effects may operate. In addition, it reinforces calls to improve parental access to health and social work professionals, through greater professional sensitivity and active outreach work, as well as diversity of provision and coordinated approaches to multiple problems (Axford, Lehtonen, Kaoukji, Tobin, & Berry, 2012; Ghate & Hazel, 2002; Whittaker & Cowley, 2012). These measures may encourage vulnerable parents to seek, and benefit from, help at an early stage, challenging negative attitudes in doing so, and facilitating fuller engagement with parenting programs designed to tackle children’s behavior problems.

## Supplementary Material

10.1037/fam0000438.supp

## Figures and Tables

**Table 1 tbl1:** Multivariable Models of Associations Between Maternal Support and Child Socioemotional Adjustment Trajectories at 70–122 Months

Measure (reference) and effect	Externalizing problems			Internalizing problems		
	Intercept	Linear slope	Quadratic slope	Intercept	Linear slope	Quadratic slope
Maternal support						
Formal support						
More	−.29***	−.01	.02*	−.32***	.01	.02
Social support						
More	−.23*	.01	.00	−.26***	−.02	.00
Covariates						
Child sex (boy)						
Girl	1.37***	.04	−.04**	.26*	.03	−.01
Developmental concern						
Yes	1.21***	−.01	−.02	1.03***	−.12*	.00
Mother’s age (30–39 years)						
<20 years	−.36	−.08	.05	−.41	−.17*	.03
20–29 years	.19	−.01	.02	.20	.00	−.01
40+ years	−.79*	−.01	.03	−.19	.04	.04
Maternal ethnic group (White)						
Minority	.58	−.11	−.02	.35	−.22*	−.08
Language at home (English)						
Other	.06	.04	.01	.41	.04	.04
Mother’s education^a^ (degree)						
Highers	.13	.02	−.01	−.38	.14	.02
Upper standard grades	.42	.04	−.04	−.33	.16**	.02
Lower standard grades or none	−.46	.01	−.02	−.69*	.12*	.02
Smoked while pregnant						
Yes	.48*	.05	−.01	−.03	.07	.00
Maternal mental health						
Better	−.03*	.00	.00	−.05***	.00*	.00
Maternal physical health						
Better	−.03**	.00	.00	−.04***	−.01**	.00
Partner relationship quality						
Better	−.41***	−.02	.00	−.34***	−.01	.01
Father in household						
Yes	.73	.06	−.02	.26	.11*	−.02
No. of children						
More	−.37***	−.01	.02*	−.35***	−.02	.01
Grandparent in household						
Yes	.64	.06	−.02	1.19*	.02	−.08
Other adult in household						
Yes	−.12	.03	.00	−1.00*	−.01	.08*
Family poverty						
Greater	.27***	.00	.00	.31***	.02	−.01
*Note.* Data presented are standardized betas, and analysis is based on 20 imputed data sets. *N* = 2649. Intercepts were set at 90 months. Model fit statistics were as follows: Comparative fit index = .98; root-mean-square error of approximation = .03; standard root-mean-square residual = .01.						
^a^ Educational qualifications are based on the Scottish Credit and Qualifications Framework and relate to the academic qualifications stated together with their vocational equivalent. Highers and standard grades are qualifications obtained by secondary school pupils. Highers allow for access to university, and standard grades at a higher (credit) or lower (general−foundation) level are typically obtained by minimum school-leaving age (16 years).						
* *p* < .05. ** *p* < .01. *** *p* < .001.						

**Table 2 tbl2:** Mediation of Associations Between Maternal Support and Child Socioemotional Adjustment Trajectory Intercepts

Variable	Externalizing problems intercept		Internalizing problems intercept	
	Not adjusted for mediators	Adjusted for mediators	Not adjusted for mediators	Adjusted for mediators
Formal support	−.29***	−.19*	−.32***	−.28***
Social support	−.23*	−.09	−.26***	−.15*
Economic strain		.14*		.21**
Maternal distress		.13		.31***
Dysfunctional parenting		1.08***		.37***
*Note.* Data presented are standardized betas, and analysis is based on 20 imputed data sets. *N* = 2649. Models are adjusted for child gender and developmental delay, maternal age, ethnic group, education, physical and mental health, smoking in pregnancy, couple relationship, father in household, number of children, grandparent in household, additional adult in household, and family poverty.				
* *p* < .05. ** *p* < .01. *** *p* < .001.				

**Table 3 tbl3:** Indirect Effects From Mother Support to Child Socioemotional Adjustment via Mediators

Support type and mediator	Externalizing problem intercept (90 months)	Internalizing problem intercept (90 months)
Formal support		
Dysfunctional parenting	−.096 [−.144, −.052]	−.036 [−.062, −.018]
Social support		
Maternal distress	*ns*	−.031 [−.062, −.009]
Maternal distress and dysfunctional parenting	−.062 [−.093, −.038]	−.023 [−.040, −.013]
Economic strain	−.011 [−.031, −.001]	−.012 [−.033, −.002]
Economic strain and maternal distress	*ns*	−.004 [−.010, −.001]
Economic strain and dysfunctional parenting	−.007 [−.015, −.002]	−.003 [−.007, −.001]
Economic strain, maternal distress, and dysfunctional parenting	−.007 [−.016, −.002]	−.003 [−.007, −.001]
*Note.* Data presented are bias-corrected bootstrapped estimates, with 95% confidence intervals in parentheses, using a nonimputed analytic sample. For simplicity, nonsignificant effects for both externalizing and internalizing problems have been omitted. Models adjusted for child gender and developmental delay, maternal age, ethnic group, education, physical and mental health, smoking in pregnancy, couple relationship, father in household, number of children, grandparent in household, additional adult in household, family poverty.		

## Footnotes

1 Although items excluded people living in the same household as the respondent, there were no mothers reporting no close family who had a grandparent or other adult (besides their partner) living with them. Only two of the 25 mothers reporting “no close friends” had a grandparent, and three had another adult besides their partner living in the same household.

2 Respondents who said they did not need any help (*n* = 104; 4.0%) were recoded as receiving enough.

## References

[c1] Axford N., Lehtonen M., Kaoukji D., Tobin K., & Berry V. (2012). Engaging parents in parenting programs: Lessons from research and practice. Children and Youth Services Review, 34, 2061–2071. 10.1016/j.childyouth.2012.06.011

[c2] Barnett M. A., Scaramella L. V., Neppl T. K., Ontai L. L., & Conger R. D. (2010). Grandmother involvement as a protective factor for early childhood social adjustment. Journal of Family Psychology, 24, 635–645. 10.1037/a0020829 20954774 PMC2976599

[c3] Barrera M. Jr. (1986). Distinctions between social support concepts, measures, and models. American Journal of Community Psychology, 14, 413–445. 10.1007/BF00922627

[c4] Belsky J. (1984). The determinants of parenting: A process model. Child Development, 55, 83–96. 10.2307/1129836 6705636

[c5] Belsky J. (1990). Parental and nonparental child-care and children’s socioemotional development: A decade in review. Journal of Marriage and Family, 52, 885–903. 10.2307/353308

[c6] Berg-Nielsen T. S., Vikan A., & Dahl A. A. (2002). Parenting related to child and parental psychopathology: A descriptive review of the literature. Clinical Child Psychology and Psychiatry, 7, 529–552. 10.1177/1359104502007004006

[c7] Berry J. O., & Jones W. H. (1995). The Parental Stress Scale: Initial psychometric evidence. Journal of Social and Personal Relationships, 12, 463–472. 10.1177/0265407595123009

[c8] Bonds D. D., Gondoli D. M., Sturge-Apple M. L., & Salem L. N. (2002). Parenting stress as a mediator of the relation between parenting support and optimal parenting. Parenting: Science and Practice, 2, 409–435. 10.1207/s15327922par0204_04

[c9] Bradshaw P., Tipping S., Marryat L., & Corbett J. (2007). Growing up in Scotland sweep 1: 2005 user guide. Edinburgh, United Kingdom: Scottish Centre for Social Research.

[c10] Bronfenbrenner U., & Morris P. A. (1998). The ecology of developmental processes In Damon W. & Lerner R. M. (Eds.), Handbook of child psychology: Theoretical models of human development (5th ed., Vol. 1, pp. 993–1028). Hoboken, NJ: Wiley.

[c11] Brown J. D., Harris S. K., Woods E. R., Buman M. P., & Cox J. E. (2012). Longitudinal study of depressive symptoms and social support in adolescent mothers. Maternal and Child Health Journal, 16, 894–901. 10.1007/s10995-011-0814-9 21556696

[c12] Choi J.-K., & Pyun H.-S. (2014). Nonresident fathers’ financial support, informal instrumental support, mothers’ parenting, and child development in single-mother families with low income. Journal of Family Issues, 35, 526–546. 10.1177/0192513X13478403

[c13] Cohen S., & Wills T. A. (1985). Stress, social support, and the buffering hypothesis. Psychological Bulletin, 98, 310–357. 10.1037/0033-2909.98.2.310 3901065

[c14] Conger R. D., Ge X., Elder G. H. Jr., Lorenz F. O., & Simons R. L. (1994). Economic stress, coercive family process, and developmental problems of adolescents. Child Development, 65, 541–561. 10.2307/1131401 8013239

[c15] Cuellar J., Jones D. J., & Sterrett E. (2015). Examining parenting in the neighborhood context: A review. Journal of Child and Family Studies, 24, 195–219. 10.1007/s10826-013-9826-y 26392738 PMC4573634

[c16] European Union (2012). Measuring material deprivation in the EU: Indicators for the whole population and child-specific indicators. Luxembourg City, Luxembourg: Publications Office of the European Union.

[c17] Fergusson D. M., Horwood L. J., & Ridder E. M. (2005). Show me the child at seven: The consequences of conduct problems in childhood for psychosocial functioning in adulthood. Journal of Child Psychology and Psychiatry, 46, 837–849. 10.1111/j.1469-7610.2004.00387.x 16033632

[c18] Ghate D., & Hazel N. (2002). Parenting in poor environments: Stress, support, and coping. London, United Kingdom: Kingsley.

[c19] Goodman A., Lamping D. L., & Ploubidis G. B. (2010). When to use broader internalising and externalising subscales instead of the hypothesised five subscales on the Strengths and Difficulties Questionnaire (SDQ): Data from British parents, teachers and children. Journal of Abnormal Child Psychology, 38, 1179–1191. 10.1007/s10802-010-9434-x 20623175

[c20] Goodman R. (1997). The Strengths and Difficulties Questionnaire: A research note. Journal of Child Psychology and Psychiatry, 38, 581–586. 10.1111/j.1469-7610.1997.tb01545.x 9255702

[c21] Harknett K. (2006). The relationship between private safety nets and economic outcomes among single mothers. Journal of Marriage and Family, 68, 172–191. 10.1111/j.1741-3737.2006.00250.x

[c22] Heberle A. E., & Carter A. S. (2015). Cognitive aspects of young children’s experience of economic disadvantage. Psychological Bulletin, 141, 723–746. 10.1037/bul0000010 25822131

[c23] Heberle A. E., Krill S. C., Briggs-Gowan M. J., & Carter A. S. (2015). Predicting externalizing and internalizing behavior in kindergarten: Examining the buffering role of early social support. Journal of Clinical Child and Adolescent Psychology, 44, 640–654. 10.1080/15374416.2014.886254 24697587 PMC4185019

[c24] Henry J. D., & Crawford J. R. (2005). The short-form version of the Depression Anxiety Stress Scales (DASS-21): Construct validity and normative data in a large non-clinical sample. British Journal of Clinical Psychology, 44, 227–239. 10.1348/014466505X29657 16004657

[c25] Herwig J. E., Wirtz M., & Bengel J. (2004). Depression, partnership, social support, and parenting: Interaction of maternal factors with behavioral problems of the child. Journal of Affective Disorders, 80, 199–208. 10.1016/S0165-0327(03)00112-5 15207933

[c26] Hu L., & Bentler P. M. (1999). Cutoff criteria for fit indexes in covariance structure analysis: Conventional criteria versus new alternatives. Structural Equation Modeling, 6, 1–55. 10.1080/10705519909540118

[c27] Hur E., Buettner C. K., & Jeon L. (2015). Parental depressive symptoms and children’s school-readiness: The indirect effect of household chaos. Journal of Child and Family Studies, 24, 3462–3473. 10.1007/s10826-015-0147-1

[c28] Jenkinson C., & Layte R. (1997). Development and testing of the UK SF-12 [short form health survey]. Journal of Health Services Research & Policy, 2, 14–18. 10.1177/135581969700200105 10180648

[c29] Kiernan K. E., & Mensah F. K. (2009). Poverty, maternal depression, family status and children’s cognitive and behavioural development in early childhood: A longitudinal study. Journal of Social Policy, 38, 569–588. 10.1017/S0047279409003250

[c30] Lakey B., & Orehek E. (2011). Relational regulation theory: A new approach to explain the link between perceived social support and mental health. Psychological Review, 118, 482–495. 10.1037/a0023477 21534704

[c31] Lee C.-Y. S., Anderson J. R., Horowitz J. L., & August G. J. (2009). Family income and parenting: The role of parental depression and social support. Family Relations, 58, 417–430. 10.1111/j.1741-3729.2009.00563.x

[c32] Lee L.-C., Halpern C. T., Hertz-Picciotto I., Martin S. L., & Suchindran C. M. (2006). Child care and social support modify the association between maternal depressive symptoms and early childhood behaviour problems: A U.S. national study. Journal of Epidemiology and Community Health, 60, 305–310. 10.1136/jech.2005.040956 16537346 PMC2593413

[c33] Leventhal T., Brooks-Gunn J., McCormick M. C., & McCarton C. M. (2000). Patterns of service use in preschool children: Correlates, consequences, and the role of early intervention. Child Development, 71, 802–819. 10.1111/1467-8624.00186 10953944

[c34] MacKinnon D. P., Lockwood C. M., & Williams J. (2004). Confidence limits for the indirect effect: Distribution of the product and resampling methods. Multivariate Behavioral Research, 39, 99–128. 10.1207/s15327906mbr3901_4 20157642 PMC2821115

[c35] Manuel J. I., Martinson M. L., Bledsoe-Mansori S. E., & Bellamy J. L. (2012). The influence of stress and social support on depressive symptoms in mothers with young children. Social Science & Medicine, 75, 2013–2020. 10.1016/j.socscimed.2012.07.034 22910191

[c36] Marshall N. L., Noonan A. E., McCartney K., Marx F., & Keefe N. (2001). It takes an urban village: Parenting networks of urban families. Journal of Family Issues, 22, 163–182. 10.1177/019251301022002003

[c37] Matheny J. A. P. Jr., Wachs T. D., Ludwig J. L., & Phillips K. (1995). Bringing order out of chaos: Psychometric characteristics of the Confusion, Hubbub, and Order Scale. Journal of Applied Developmental Psychology, 16, 429–444. 10.1016/0193-3973(95)90028-4

[c38] Maupin A. N., Brophy-Herb H. E., Schiffman R. F., & Bocknek E. L. (2010). Low-income parental profiles of coping, resource adequacy, and public assistance receipt: Links to parenting. Family Relations, 59, 180–194. 10.1111/j.1741-3729.2010.00594.x

[c39] McConnell D., Breitkreuz R., & Savage A. (2011). From financial hardship to child difficulties: Main and moderating effects of perceived social support. Child: Care, Health and Development, 37, 679–691. 10.1111/j.1365-2214.2010.01185.x 21143271

[c40] Muthén L. K., & Muthén B. O. (1998–2012). Mplus user’s guide (7th ed.). Los Angeles, CA: Author.

[c41] Östberg M., & Hagekull B. (2013). Parenting stress and external stressors as predictors of maternal ratings of child adjustment. Scandinavian Journal of Psychology, 54, 213–221. 10.1111/sjop.12045 23480459

[c42] Pianta R. C. (1992). Child-Parent Relationship Scale. Unpublished measure, Curry School of Education, University of Virginia, Charlottesville, VA. 10.3389/fpsyg.2023.1194644 PMC10547905 37799528

[c43] Rust J., Bennun I., Crowe M., & Golombok S. (1990). The GRIMS: A psychometric instrument for the assessment of marital discord. Journal of Family Therapy, 12, 45–57. 10.1046/j.1990.00369.x

[c44] Ryan R. M., Kalil A., & Leininger L. (2009). Low-income mothers’ private safety nets and children’s socioemotional well-being. Journal of Marriage and Family, 71, 278–297. 10.1111/j.1741-3737.2008.00599.x

[c45] Ryan R. M., Tolani N., & Brooks-Gunn J. (2009). Relationship trajectories, parenting stress, and unwed mothers’ transition to a new baby. Parenting, Science and Practice, 9, 160–177. 10.1080/15295190802656844 PMC2843930 20333272

[c46] Sayal K., Washbrook E., & Propper C. (2015). Childhood behavior problems and academic outcomes in adolescence: Longitudinal population-based study. Journal of the American Academy of Child & Adolescent Psychiatry, 54, 360–368.e2. 10.1016/j.jaac.2015.02.007 25901772

[c47] Shonkoff J. P., Garner A. S., & the Committee on Psychosocial Aspects of Child and Family Health, Committee on Early Childhood, Adoption, and Dependent Care, & the Section on Developmental and Behavioral Pediatrics (2012). The lifelong effects of early childhood adversity and toxic stress. Pediatrics, 129, e232–e246. 10.1542/peds.2011-2663 22201156

[c48] Spielberger J., & Lyons S. J. (2009). Supporting low-income families with young children: Patterns and correlates of service use. Children and Youth Services Review, 31, 864–872. 10.1016/j.childyouth.2009.03.009

[c49] Stone L. L., Otten R., Engels R. C., Vermulst A. A., & Janssens J. M. (2010). Psychometric properties of the parent and teacher versions of the Strengths and Difficulties Questionnaire for 4- to 12-year-olds: A review. Clinical Child and Family Psychology Review, 13, 254–274. 10.1007/s10567-010-0071-2 20589428 PMC2919684

[c50] Thoits P. A. (2011). Mechanisms linking social ties and support to physical and mental health. Journal of Health and Social Behavior, 52, 145–161. 10.1177/0022146510395592 21673143

[c51] Weeks M., Ploubidis G. B., Cairney J., Wild T. C., Naicker K., & Colman I. (2016). Developmental pathways linking childhood and adolescent internalizing, externalizing, academic competence, and adolescent depression. Journal of Adolescence, 51, 30–40. 10.1016/j.adolescence.2016.05.009 27288965

[c52] Wetherby A. M., Allen L., Cleary J., Kublin K., & Goldstein H. (2002). Validity and reliability of the Communication and Symbolic Behavior Scales Developmental Profile with very young children. Journal of Speech, Language, and Hearing Research, 45, 1202–1218. 10.1044/1092-4388(2002/097) 12546488

[c53] Whittaker K. A., & Cowley S. (2012). An effective programme is not enough: A review of factors associated with poor attendance and engagement with parenting support programmes. Children & Society, 26, 138–149. 10.1111/j.1099-0860.2010.00333.x

