# Supplementary material for: Direct, Indirect, and Buffering Effects of Support for Mothers on Children’s Socioemotional Adjustment
Source: J Fam Psychol. 2018 Aug 9;32(7):894–903. doi: 10.1037/fam0000438 (PMC6205417; doi:10.1037/fam0000438)
Supplement: Supplementary file 1 [file FAM-2017-1171Supp.zip › Final Revision 1 Online Resource 1 covariates.pdf]

Online Resource 1 Information on covariate measures in the analytic sample, n=2649

Measure	Time point	Response category/ range	% or mean (standard error)
**Child characteristics**			
Gender		male	51.6
Developmental delay	22 months	yes	7.3
**Maternal characteristics**			
Age at birth of cohort child	10 months	<20 years	7.2
		20-29 years	41.5
		30-39 years	48.2
		40+ years	3.1
Ethnic group	10 months	Minority	2.9
Language spoken at home	10 months	Language other than English	4.6
Education[a]	10 months	Degree-level	25.7
		Highers	33.6
		Upper Standard grades	25.7
		Lower Standard grades	6.4
		No qualifications	8.8
Smoked while pregnant	10 months	yes	24.1
Mental health	10 months	10.12 to 66.24	50.04 (0.25)
Physical health	10 months	14.93 to 66.00	53.27 (0.17)
Partner relationship quality	22 months	22 months	0.02 (0.03)
**Household characteristics**			
Resident father	10 months	not resident	20.6
Adults in household (in addition to parents)	10 months	grandparent(s)	5.8
		other adult(s)	7.8
Number of children	10 months	one	48.7
		two	33.9
		three	13.2
		four or more	4.2
Poverty score	10-22 months		0.97 (0.07)

Note: figures shown take account of complex survey design and survey weights. [a]Scottish national educational qualifications: at the time of the survey, Standard grades and Highers were qualifications typically obtained in the fourth and fifth year of secondary school. Upper level standard grades refers to grades 1-3, lower level to grades 4-6.
