# Supplementary material for: Direct, Indirect, and Buffering Effects of Support for Mothers on Children’s Socioemotional Adjustment
Source: J Fam Psychol. 2018 Aug 9;32(7):894–903. doi: 10.1037/fam0000438 (PMC6205417; doi:10.1037/fam0000438)
Supplement: Supplementary file 1 [file FAM-2017-1171Supp.zip › Final Revision 1 Online Resource 2 correlations.pdf]

Online Resource 2 Sample information and zero-order correlations for measures of child socio-emotional adjustment, maternal support and mediators (n=2649)

		Mean(SE)	1	2	3	4	5	6	7	8	9	10	11	12	13
1	Formal support	-0.12 (0.03)	1.00												
2	Social support	-0.03 (0.03)	0.17	1.00											
3	Maternal distress	0.10 (0.03)	-0.18	-0.27	1.00										
4	Economic hardship	0.18 (0.04)	-0.20	-0.13	0.37	1.00									
5	Dysfunctional parenting	0.06 (0.03)	-0.20	-0.22	0.52	0.26	1.00								
6	Externalising problems 58 months	5.51 (0.09)	-0.18	-0.12	0.30	0.23	0.45	1.00							
7	Externalising problems 70 months	5.18 (0.09)	-0.15	-0.15	0.35	0.24	0.53	0.70	1.00						
8	Externalising problems 94 months	5.09 (0.08)	-0.17	-0.14	0.29	0.23	0.46	0.66	0.74	1.00					
9	Externalising problems 122 months	4.44 (0.09)	-0.17	-0.12	0.26	0.24	0.40	0.60	0.65	0.74	1.00				
10	Internalising problems 58 months	2.35 (0.07)	-0.17	-0.15	0.26	0.20	0.25	0.29	0.26	0.26	0.23	1.00			
11	Internalising problems 70 months	2.31 (0.08)	-0.17	-0.15	0.28	0.21	0.32	0.27	0.33	0.29	0.25	0.61	1.00		
12	Internalising problems 94 months	2.77 (0.10)	-0.19	-0.17	0.29	0.22	0.28	0.27	0.31	0.38	0.30	0.53	0.64	1.00	
13	Internalising problems 122 months	3.02 (0.10)	-0.20	-0.17	0.29	0.28	0.29	0.30	0.32	0.39	0.45	0.46	0.56	0.61	1.00

Note: SE=standard error. All correlations $p<0.001$.
