# Supplementary material for: Direct, Indirect, and Buffering Effects of Support for Mothers on Children’s Socioemotional Adjustment
Source: J Fam Psychol. 2018 Aug 9;32(7):894–903. doi: 10.1037/fam0000438 (PMC6205417; doi:10.1037/fam0000438)
Supplement: Supplementary file 1 [file FAM-2017-1171Supp.zip › Final Revision 1 Online Resource 1 covariates.docx]

Online Resource 1 Information on covariate measures in the analytic sample, n=2649

| **Measure** | **Time point** | **Response category/**  **range** | **% or mean (standard error)** |
| --- | --- | --- | --- |
| **Child characteristics** |  |  |  |
| Gender |  | male | 51.6 |
| Developmental delay | 22 months | yes | 7.3 |
| **Maternal characteristics** |  |  |  |
| Age at birth of cohort child | 10 months | <20 years | 7.2 |
|  |  | 20-29 years | 41.5 |
|  |  | 30-39 years | 48.2 |
|  |  | 40+ years | 3.1 |
| Ethnic group | 10 months | Minority | 2.9 |
| Language spoken at home | 10 months | Language other than English | 4.6 |
| Education^a^ | 10 months | Degree-level | 25.7 |
|  |  | Highers | 33.6 |
|  |  | Upper Standard grades | 25.7 |
|  |  | Lower Standard grades | 6.4 |
|  |  | No qualifications | 8.8 |
| Smoked while pregnant | 10 months | yes | 24.1 |
| Mental health | 10 months | 10.12 to 66.24 | 50.04 (0.25) |
| Physical health | 10 months | 14.93 to 66.00 | 53.27 (0.17) |
| Partner relationship quality | 22 months | 22 months | 0.02 (0.03) |
| **Household characteristics** |  |  |  |
| Resident father | 10 months | not resident | 20.6 |
| Adults in household (in addition to parents) | 10 months | grandparent(s) | 5.8 |
|  |  | other adult(s) | 7.8 |
| Number of children | 10 months | one | 48.7 |
|  |  | two | 33.9 |
|  |  | three | 13.2 |
|  |  | four or more | 4.2 |
| Poverty score | 10-22 months |  | 0.97 (0.07) |

Note: figures shown take account of complex survey design and survey weights. ^a^Scottish national educational qualifications: at the time of the survey, Standard grades and Highers were qualifications typically obtained in the fourth and fifth year of secondary school. Upper level standard grades refers to grades 1-3, lower level to grades 4-6.
