# Supplementary material for: Direct, Indirect, and Buffering Effects of Support for Mothers on Children’s Socioemotional Adjustment
Source: J Fam Psychol. 2018 Aug 9;32(7):894–903. doi: 10.1037/fam0000438 (PMC6205417; doi:10.1037/fam0000438)
Supplement: Supplementary file 1 [file FAM-2017-1171Supp.zip › Final Revision 1 Online Resource 2 correlations.docx]

Online Resource 2 Sample information and zero-order correlations for measures of child socio-emotional adjustment, maternal support and mediators (n=2649)

|  |  |  | 1 | 2 | 3 | 4 | 5 | 6 | 7 | 8 | 9 | 10 | 11 | 12 | 13 |
| --- | --- | --- | --- | --- | --- | --- | --- | --- | --- | --- | --- | --- | --- | --- | --- |
|  |  | Mean(SE) |  |  |  |  |  |  |  |  |  |  |  |  |  |
| 1 | Formal support | -0.12 (0.03) | 1.00 |  |  |  |  |  |  |  |  |  |  |  |  |
| 2 | Social support | -0.03 (0.03) | 0.17 | 1.00 |  |  |  |  |  |  |  |  |  |  |  |
| 3 | Maternal distress | 0.10 (0.03) | -0.18 | -0.27 | 1.00 |  |  |  |  |  |  |  |  |  |  |
| 4 | Economic hardship | 0.18 (0.04) | -0.20 | -0.13 | 0.37 | 1.00 |  |  |  |  |  |  |  |  |  |
| 5 | Dysfunctional parenting | 0.06 (0.03) | -0.20 | -0.22 | 0.52 | 0.26 | 1.00 |  |  |  |  |  |  |  |  |
| 6 | Externalising problems 58 months | 5.51 (0.09) | -0.18 | -0.12 | 0.30 | 0.23 | 0.45 | 1.00 |  |  |  |  |  |  |  |
| 7 | Externalising problems 70 months | 5.18 (0.09) | -0.15 | -0.15 | 0.35 | 0.24 | 0.53 | 0.70 | 1.00 |  |  |  |  |  |  |
| 8 | Externalising problems 94 months | 5.09 (0.08) | -0.17 | -0.14 | 0.29 | 0.23 | 0.46 | 0.66 | 0.74 | 1.00 |  |  |  |  |  |
| 9 | Externalising problems 122 months | 4.44 (0.09) | -0.17 | -0.12 | 0.26 | 0.24 | 0.40 | 0.60 | 0.65 | 0.74 | 1.00 |  |  |  |  |
| 10 | Internalising problems 58 months | 2.35 (0.07) | -0.17 | -0.15 | 0.26 | 0.20 | 0.25 | 0.29 | 0.26 | 0.26 | 0.23 | 1.00 |  |  |  |
| 11 | Internalising problems 70 months | 2.31 (0.08) | -0.17 | -0.15 | 0.28 | 0.21 | 0.32 | 0.27 | 0.33 | 0.29 | 0.25 | 0.61 | 1.00 |  |  |
| 12 | Internalising problems 94 months | 2.77 (0.10) | -0.19 | -0.17 | 0.29 | 0.22 | 0.28 | 0.27 | 0.31 | 0.38 | 0.30 | 0.53 | 0.64 | 1.00 |  |
| 13 | Internalising problems 122 months | 3.02 (0.10) | -0.20 | -0.17 | 0.29 | 0.28 | 0.29 | 0.30 | 0.32 | 0.39 | 0.45 | 0.46 | 0.56 | 0.61 | 1.00 |

Note: SE=standard error. All correlations *p*<0.001.
