# Supplementary material for: Direct, Indirect, and Buffering Effects of Support for Mothers on Children’s Socioemotional Adjustment
Source: J Fam Psychol. 2018 Aug 9;32(7):894–903. doi: 10.1037/fam0000438 (PMC6205417; doi:10.1037/fam0000438)
Supplement: Supplementary file 1 [file FAM-2017-1171Supp.zip › Final Revision 1 Online Resource 3 graphs.docx]

Online Resource 3
Graph of predicted trajectories of child socio-emotional adjustment from age 48 to 122 months
